# BMI and Prognostic Nutritional Index Are Independently and Positively Associated with Three Year Glycemic Change in Non-Diabetic Adults: A Community-Based Cohort Study

**DOI:** 10.3390/nu18091459

**Published:** 2026-05-01

**Authors:** Yuting Yu, Li Chen, Wei Zhang, Lihua Jiang, Chunmin Zhang, Xiaoying Ni, Jianguo Yu, Yonggen Jiang

**Affiliations:** 1 Songjiang District Center for Disease Control and Prevention and Health Inspection, Shanghai 201600, China; 19111020017@fudan.edu.cn; 2 Zhongshan Community Health Center, Shanghai 201613, China; weiwan1120@163.com (L.C.); 18017908596@163.com (W.Z.); 3 Sheshan Community Health Center, Shanghai 201602, China; jianglihua2000@126.com; 4 Xinqiao Community Health Center, Shanghai 201612, China; zhangchunmin268@163.com; 5 Maogang Community Health Center, Shanghai 201607, China; nixiaoying2026@163.com

**Keywords:** body mass index, prognostic nutritional index, glycemic change, HbA1c, cohort study

## Abstract

**Background/Objectives**: Both adiposity and nutritional–inflammatory status influence glucose metabolism; however, their longitudinal associations with glycemic changes in non-diabetic populations remain unclear. We examined the independent, interactive, and joint associations of body mass index (BMI) and prognostic nutritional index (PNI) with the 3-year change in HbA1c (ΔHbA1c). PNI, a composite marker of serum albumin and peripheral lymphocyte count, reflects both protein nutritional status and systemic immune competence. We hypothesized that BMI and PNI would each independently predict ΔHbA1c and that their joint profiling would identify higher-risk subgroups. **Methods**: A total of 9414 non-diabetic adults from the Shanghai Suburban Adult Cohort were included. Participants with diabetes at baseline (defined as fasting plasma glucose ≥ 7.0 mmol/L, 2-h post-load glucose ≥ 11.1 mmol/L, HbA1c ≥ 6.5%, or self-reported physician diagnosis of diabetes or use of glucose-lowering medications) were excluded. BMI was measured, and PNI was calculated as serum albumin + 5 × lymphocyte count. ΔHbA1c was assessed over a 3-year period. Multivariable linear regression, interaction testing, and joint stratification were performed. Covariate selection was guided by prior biological plausibility, and model adequacy was evaluated using the Akaike Information Criterion (AIC). **Results**: Both BMI (β = 0.013% per kg/m^2^, 95% CI: 0.011–0.016, *p* < 0.001) and PNI (β = 0.002% per unit, 95% CI: 0.000–0.004, *p* = 0.019) were independently and positively associated with ΔHbA1c. No significant interaction was observed (*p* = 0.431). High BMI (≥24 kg/m^2^) was associated with glycemic worsening irrespective of PNI level (β ≈ 0.075%, *p* < 0.001). Among normal-weight individuals, higher PNI was associated with a modest increase in ΔHbA1c (β = 0.031%, *p* = 0.007). **Conclusions**: Although the absolute effect sizes were modest at the individual level, BMI was consistently and independently associated with glycemic deterioration therefore, even small per-unit increases may translate into meaningful risk at the population level given the high prevalence of overweight and obesity. PNI showed a small positive association, suggesting that in relatively healthy populations a higher PNI may partly capture subtle pro-glycemic factors—such as low-grade inflammation or higher protein intake—rather than representing unambiguous nutritional benefit. The absence of interaction suggests that BMI and PNI act through largely independent pathways. These findings extend prior evidence by demonstrating that PNI provides modest additional glycemic information beyond BMI in non-diabetic community-dwelling adults, particularly among those of normal weight.

## 1. Introduction

The global prevalence of type 2 diabetes mellitus (T2DM) continues to rise, particularly in low- and middle-income countries, underscoring the urgent need to identify modifiable risk factors and early metabolic alterations [1,2]. Glycemic deterioration, reflected by increases in hemoglobin A1c (HbA1c) over time, often precedes the clinical diagnosis of diabetes and represents a critical window for preventive intervention [3]. Understanding the factors associated with such deterioration in non-diabetic populations is therefore of major public health importance.

Obesity, commonly assessed by body mass index (BMI), is a well-established risk factor for insulin resistance and T2DM [4]. Numerous studies have demonstrated a positive association between BMI and longitudinal increases in HbA1c, even within the normal range [5]. However, the magnitude of this association may vary across individuals, suggesting the presence of effect modifiers [6].

Nutritional status, beyond its contribution to body weight, has emerged as a potential modulator of glucose metabolism. The prognostic nutritional index (PNI), originally proposed by Buzby et al. [7] to predict surgical outcomes, is calculated as: PNI = serum albumin (g/L) + 5 × peripheral blood lymphocyte count (×10^9^/L). It serves as a composite indicator of both protein nutritional reserve and systemic immune competence [8]. Serum albumin reflects long-term protein intake and hepatic synthetic function, while lymphocyte count captures cell-mediated immunity, which is closely linked to nutritional adequacy [7]. A higher PNI is generally interpreted as indicating better protein stores and immune function, with lower values associated with malnutrition and increased risk of adverse outcomes [7,8]. Beyond surgical settings, PNI has been increasingly applied in metabolic research. Recent studies have found inverse associations between PNI and prevalent T2DM and its complications [9,10], suggesting that poor nutritional–immune status may contribute to dysglycemia. However, the longitudinal relationship between PNI and glycemic changes in healthy, non-diabetic populations remains underexplored.

Among the various available nutritional indicators—including the Geriatric Nutritional Risk Index (GNRI), the Nutritional Risk Index (NRI), and the Controlling Nutritional Status (CONUT) score—we selected PNI for several reasons. First, PNI requires only two routinely available laboratory parameters (serum albumin and lymphocyte count), making it highly practical in community health settings. Second, unlike composite scores that incorporate BMI (e.g., GNRI), PNI is independent of body weight, enabling its simultaneous evaluation alongside BMI without collinearity concerns. Third, existing evidence links PNI to glycemic and inflammatory outcomes [9,10], providing a biologically plausible rationale for its investigation in this context.

Given that both BMI and PNI reflect distinct but interconnected aspects of metabolic health—adiposity and nutrition/immunity—their joint effects on glycemic trajectories warrant investigation. It is plausible that nutritional–immune status may modify the impact of obesity on glucose homeostasis [11]. For instance, individuals with high BMI but well-preserved protein stores and immune function (high PNI) may exhibit different glycemic trajectories compared with those with high BMI and compromised nutritional–immune status (low PNI). Conversely, in normal-weight individuals, nutritional–immune status may play a more prominent role in metabolic regulation [12].

To date, few studies have comprehensively examined the independent, interactive, and joint associations of BMI and PNI with longitudinal glycemic changes in a general population without diabetes. The present study addresses this gap using a large community-based prospective cohort. Compared with prior cross-sectional studies that cannot disentangle cause from effect, our longitudinal design allows temporal inference. Furthermore, by simultaneously evaluating both BMI and PNI—rather than each in isolation—we provide a more complete picture of how adiposity and nutritional–immune status together shape glycemic trajectories. This dual profiling approach may help identify higher-risk subgroups and inform more targeted preventive strategies.

Therefore, we aimed to: (1) investigate the independent associations of baseline BMI and PNI with 3-year changes in HbA1c (ΔHbA1c) among Chinese adults without diabetes; (2) examine whether PNI modifies the association between BMI and ΔHbA1c; and (3) explore the combined effects of BMI and PNI categories on glycemic deterioration. We hypothesized that higher BMI would be associated with greater ΔHbA1c, and that PNI would provide additional glycemic predictive information, particularly among normal-weight individuals, potentially acting as a surrogate for nutritional–inflammatory factors that influence β-cell function and insulin sensitivity.

## 2. Materials and Methods

### 2.1. Study Design and Population

This cohort study utilized data from the Shanghai Suburban Adult Cohort and Biobank (SSACB) [13], a community-based prospective cohort jointly established by the School of Public Health, Fudan University, and the Shanghai Songjiang Center for Disease Control and Prevention. Baseline examinations were conducted between June 2016 and December 2017, with follow-up examinations performed between June 2019 and December 2020, providing a median follow-up duration of 3 years.

For the present analysis, participants were eligible if they met the following criteria at baseline: (1) aged 20–74 years; (2) free of diabetes at baseline, defined as fasting plasma glucose < 7.0 mmol/L, 2-h post-load glucose < 11.1 mmol/L, HbA1c < 6.5%, and no self-reported physician diagnosis of diabetes or use of glucose-lowering medications; and (3) complete data on exposure variables (BMI and PNI components), outcomes (HbA1c at both baseline and follow-up), and key covariates.

Participants were excluded if they had: (1) diabetes at baseline as defined above (i.e., meeting any one of the above glycemic thresholds, or reporting a physician diagnosis of diabetes, or using glucose-lowering medications); (2) missing HbA1c measurements at either baseline or follow-up; (3) missing data on serum albumin or lymphocyte count required for PNI calculation; (4) missing data on key covariates; (5) severe comorbidities that could independently affect nutritional status or glycemic metabolism, including active cancer, liver cirrhosis, or end-stage renal disease; or (6) acute or chronic infections or immune-mediated inflammatory diseases (e.g., autoimmune disorders, known chronic inflammatory conditions) at baseline, as these conditions directly alter lymphocyte counts and serum albumin levels and would therefore confound PNI interpretation. A total of 9414 participants were included in the final analysis.

To address potential residual confounding by medications and comorbidities, we additionally recorded baseline use of antihypertensive medications and lipid-lowering drugs. Participants taking these medications were not excluded, as exclusion would have introduced selection bias toward healthier individuals; instead, systolic and diastolic blood pressure and lipid profiles—which mediate or reflect the effects of these medications—were included as covariates in all adjusted models. Participants with a history of cardiovascular or cerebrovascular disease at baseline were retained in the main analysis but were excluded in a sensitivity analysis to verify the robustness of the primary findings.

The baseline cohort included both normoglycemic individuals (HbA1c < 5.7%) and those with prediabetes (HbA1c 5.7–6.4%), consistent with standard clinical categories. Given that the effects of BMI and PNI may differ between these groups, we conducted pre-specified subgroup analyses stratified by baseline glycemic status (normoglycemic vs. prediabetes) to examine whether the primary associations were consistent across these subgroups.

### 2.2. Exposure Assessment

Body weight was measured to the nearest 0.1 kg using a calibrated electronic scale (seca 876, seca GmbH & Co. KG, Hamburg, Germany), and height was measured to the nearest 0.1 cm using a wall-mounted stadiometer (seca 213, seca GmbH & Co. KG, Hamburg, Germany). Blood pressure was measured using a validated electronic sphygmomanometer (HBP-1300, Omron Healthcare Co., Ltd., Kyoto, Japan). Fasting plasma glucose, total cholesterol, triglycerides, HDL cholesterol, and LDL cholesterol were quantified by standardized enzymatic methods on a Cobas C702 automated biochemical analyzer (Roche Diagnostics, Indianapolis, IN, USA). HbA1c was measured at both baseline and follow-up by high-performance liquid chromatography (HPLC) using a D-10 analyzer (Bio-Rad Laboratories, Inc., Hercules, CA, USA). Serum albumin was measured using a colorimetric assay on a Cobas C702 automated analyzer (Roche Diagnostics, Indianapolis, IN, USA), and five-part differential complete blood counts were analyzed on XS-500i and XN-1000 hematology analyzers (Sysmex Corporation, Kobe, Japan). All statistical analyses were performed using R software (version 4.2.1; R Foundation for Statistical Computing, Vienna, Austria).

#### 2.2.1. Body Mass Index (BMI)

Body weight and height were measured by trained staff using standardized protocols. Weight was measured to the nearest 0.1 kg, with participants wearing light indoor clothing and no shoes. Height was measured to the nearest 0.1 cm using a stadiometer. BMI [14] was calculated as weight in kilograms divided by height in meters squared (kg/m^2^) and was treated as a continuous variable.

#### 2.2.2. Prognostic Nutritional Index (PNI)

Fasting blood samples were collected at baseline after at least 8 h of fasting. Serum albumin levels were measured using a colorimetric assay on an automated clinical chemistry platform (C702, Roche Diagnostics, Indianapolis, IN, USA). Lymphocyte counts were derived from five-part differential complete blood counts analyzed using electrical impedance-based hematology analyzers (XS-500i and XN-1000, Sysmex Corporation, Kobe, Japan).

The PNI was calculated using the standard formula [7]:

PNI = serum albumin (g/L) + 5 × peripheral blood lymphocyte count (×10^9^/L)

The formula assigns a fivefold weight to lymphocyte count relative to albumin, reflecting the empirical observation that lymphocyte count contributes proportionally more to predicting nutritional–immune reserve in clinical validation studies [7]. Albumin captures long-term protein nutritional status (half-life ~21 days), while lymphocytes reflect the immune competence that depends on adequate micronutrient and protein availability. Together, these two components make PNI a pragmatic composite of nutritional and immune function, applicable in community settings where more detailed dietary assessments are unavailable.

PNI was analyzed both as a continuous variable and as quartiles [15,16] based on the distribution of the study population.

### 2.3. Outcome Measurement

The primary outcome was the 3-year change in glycated hemoglobin (ΔHbA1c), defined as:

ΔHbA1c (%) = HbA1c at follow-up − HbA1c at baseline

HbA1c was measured using high-performance liquid chromatography (HPLC) at both time points under standardized laboratory procedures. A positive ΔHbA1c value indicates glycemic deterioration over the follow-up period. ΔHbA1c was primarily analyzed as a continuous variable.

We chose ΔHbA1c as the primary outcome rather than incident diabetes for the following reasons. First, glycemic deterioration—even within the non-diabetic range—is a clinically meaningful intermediate endpoint that precedes and predicts future diabetes diagnosis [3]. Second, using incident diabetes as the primary endpoint in a 3-year follow-up of a community sample without diabetes would yield a relatively small number of events, limiting statistical power to detect modest associations such as those expected for PNI. Third, a continuous outcome retains more statistical information and allows detection of graded dose–response relationships. Incident diabetes and HbA1c progression to ≥6.5% were, however, examined as secondary outcomes in sensitivity analyses.

### 2.4. Covariates

At baseline, trained interviewers collected demographic characteristics, lifestyle factors, and medical history using a structured questionnaire. The following covariates were considered potential confounders:

**Demographics** [17]: Age (years, continuous) and sex (male/female).

**Socioeconomic status**: Education [18] (low/middle/high) and marital status (married/other) [19].

**Lifestyle factors**: Smoking status [20] (current smoker: ≥1 cigarette/day for >6 months; yes/no), alcohol consumption [21] (current drinker: >3 drinking occasions/week for ≥6 months; yes/no), and physical activity [22] (total metabolic equivalent of task [MET] minutes per week, continuous). MET-minutes/week were calculated based on the Compendium of Physical Activities [23].

**Anthropometrics and blood pressure**: BMI [24] (kg/m^2^, continuous) and systolic and diastolic blood pressure (mmHg, continuous variables). Blood pressure [25] was measured three times at 5-min intervals after at least 5 min of rest, and the average of the last two measurements was used.

**Biochemical measurements**: Fasting plasma glucose, HbA1c, total cholesterol, triglycerides, HDL cholesterol, and LDL cholesterol [25], measured using standardized enzymatic methods.

**Family history**: Family history of diabetes in first-degree relatives (yes/no) [26].

All covariates were selected a priori based on biological plausibility and prior literature, not through stepwise data-driven procedures. This approach minimizes the risk of overfitting while ensuring that important confounders are controlled for. Model adequacy was assessed using the Akaike Information Criterion (AIC); the fully adjusted model (Model 3) achieved lower AIC than more parsimonious models, indicating that the additional covariates improved model fit. Variance inflation factors (VIFs) were also computed to assess multicollinearity; all VIF values were below 5, confirming the absence of problematic collinearity among the 16 covariates.

### 2.5. Statistical Analysis

#### 2.5.1. Descriptive Statistics

Baseline characteristics were summarized as mean ± standard deviation (SD) for normally distributed variables, median (interquartile range, IQR) for skewed variables, and counts (percentages) for categorical variables. Normality was assessed using the Shapiro–Wilk test and histogram inspection.

#### 2.5.2. Main Analysis

Multivariable linear regression models were constructed with three progressively adjusted models (Model 1: unadjusted; Model 2: adjusted for age and sex; Model 3: fully adjusted as described above). Results are presented as β coefficients with 95% confidence intervals (CIs) and corresponding *p*-values.

#### 2.5.3. Interaction and Stratified Analyses

To test whether the association between BMI and ΔHbA1c varied by PNI level, a multiplicative interaction term (BMI × PNI) was added to the fully adjusted model. Participants were also stratified by PNI quartiles (Q1: ≤55.9; Q2: 56.0–58.7; Q3: 58.8–61.4; Q4: ≥61.5), and within each quartile the BMI–ΔHbA1c association was examined using the fully adjusted model.

#### 2.5.4. Joint Associations Analysis

Participants were cross-classified into four groups based on BMI category (normal: <24 kg/m^2^; high: ≥24 kg/m^2^, using Chinese criteria for overweight/obesity) and PNI category (low: below median; high: above median). The “Normal BMI + Low PNI” group served as the reference. This joint classification provides a clinically intuitive risk stratification framework. Adjusted mean ΔHbA1c with 95% CIs was estimated for each group, adjusting for all Model 3 covariates.

#### 2.5.5. Supplementary and Sensitivity Analyses

Non-linearity was examined using restricted cubic spline (RCS) regression [27]. Subgroup analyses were conducted stratified by age (<60 vs. ≥60 years), baseline glycemic status (normoglycemic: HbA1c < 5.7% vs. prediabetes: HbA1c 5.7–6.4%), and BMI category. Dose–response analysis was performed using simple slope analysis at selected PNI percentiles [28]. A sensitivity analysis excluding participants with a history of cardiovascular or cerebrovascular disease was also performed. Additionally, a secondary analysis examined incident diabetes (HbA1c progression to ≥6.5% or new physician diagnosis) as an outcome [29].

#### 2.5.6. Software and Significance Level

All analyses were performed using R software (version 4.2.1). A two-sided *p*-value < 0.05 was considered statistically significant.

### 2.6. Ethical Considerations

The study was approved by the Ethical Review Committee of the School of Public Health, Fudan University (IRB No. 2016-04-0586, 2 September 2024). All participants provided written informed consent. The study adhered to the Declaration of Helsinki.

## 3. Results

### 3.1. Baseline Characteristics of the Study Population

A total of 9414 participants without diabetes at baseline were included in the primary analysis. Baseline characteristics of the study population are summarized in Table 1. The mean age of participants was 56.54 years (SD 10.33), and 61% were female. The mean BMI was 24.29 kg/m^2^ (SD 3.25), and the mean PNI was 58.79 (SD 4.20). The mean baseline HbA1c was 5.62% (SD 0.40%), and the mean 3-year change in HbA1c (ΔHbA1c) was 0.35% (SD 0.62%). Of the 9414 participants, 3821 (40.6%) were normoglycemic (HbA1c < 5.7%) and 5593 (59.4%) had prediabetes (HbA1c 5.7–6.4%) at baseline.

Regarding lifestyle factors, 22% of participants were current smokers and 13% were current drinkers, while the mean physical activity (MET-min/week) was 1732.53 (SD 1165.68). Education levels were distributed as low (47%), middle (45%), and high (7.1%). Most participants were married (94%), and 9.9% reported a family history of diabetes.

### 3.2. Associations of BMI and PNI with Glycemic Change

Table 2 presents the linear regression results. In the fully adjusted model (Model 3), each 1 kg/m^2^ increase in BMI was associated with a 0.013% increase in ΔHbA1c (95% CI: 0.011–0.016; *p* < 0.001). To illustrate the practical magnitude, a 5 kg/m^2^ increase in BMI—a clinically substantial change—would be associated with a 0.067% increase in ΔHbA1c, bringing the average participant from a baseline of 5.62% to approximately 5.69% over three years. While this represents a modest absolute change at the individual level, given that the mean ΔHbA1c across the entire cohort was only 0.35%, BMI explained a meaningful proportion of the observed glycemic trajectory.

For PNI, each 1-unit increase was associated with a **0.002%** increase in ΔHbA1c (95% CI: 0.000–0.004; *p* = 0.019). The effect of PNI was considerably smaller than that of BMI, and its practical significance should be interpreted accordingly.

The interaction term between BMI and PNI was not statistically significant (β = −0.000, 95% CI: −0.001 to 0.000; *p* = 0.431).

### 3.3. Stratified Analyses by PNI Quartiles

**Figure 1:**
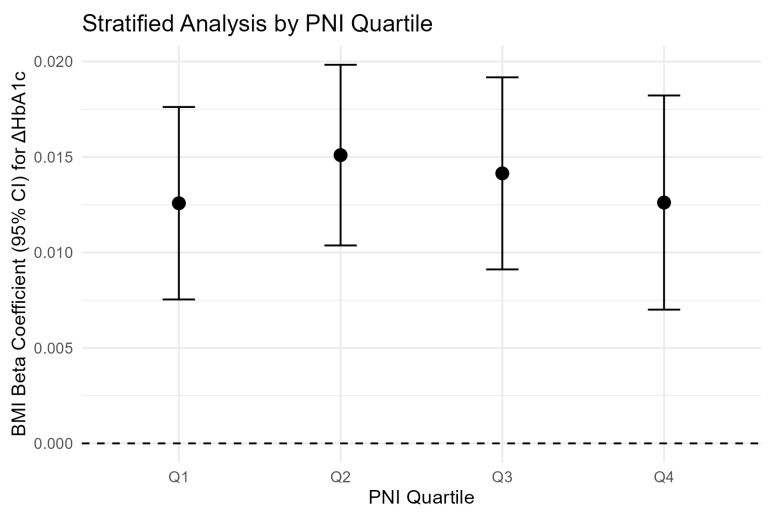
Forest plot of BMI effect on ΔHbA1c across PNI quartiles. Adjusted β coefficients (95% CI) for the association between BMI and ΔHbA1c across quartiles of PNI. Models adjusted for all covariates as in Table 3. No significant variation in effect size is observed across quartiles.

The association between BMI and ΔHbA1c was consistent across all PNI quartiles (Table 3). The covariate adjustment in Table 3 follows Model 3 (fully adjusted), with PNI treated as the stratification variable rather than as a covariate; BMI was the exposure variable within each stratum. Effect estimates ranged from 0.013 (Q1, Q4) to 0.015 (Q2), with no statistically significant trend across quartiles, confirming the absence of interaction (Figure 1).

All models adjusted for age, sex, smoking, alcohol consumption, physical activity, blood pressure, lipids, education, marital status, family history of diabetes, and baseline HbA1c. PNI is the stratification variable.

### 3.4. Joint Associations of BMI and PNI with Glycemic Change

**Figure 2:**
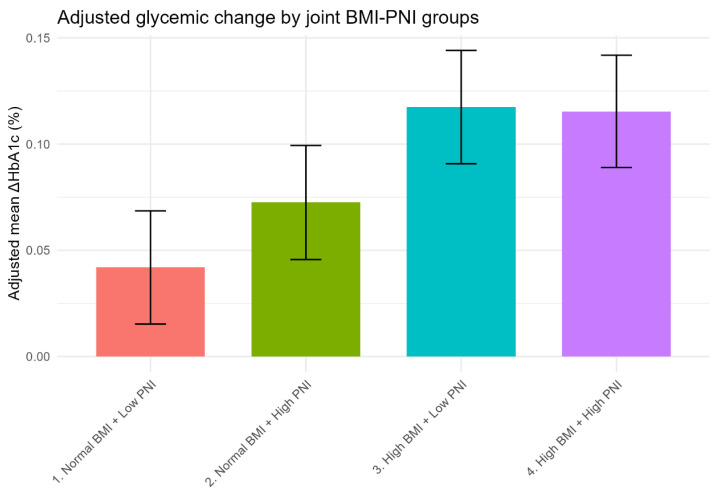
Adjusted mean ΔHbA1c by joint BMI-PNI groups. Adjusted mean ΔHbA1c (with 95% CI) for four groups defined by BMI (<24 vs. ≥24 kg/m^2^) and PNI (below vs. above median). Estimates are derived from a linear regression model including the joint group variable and all covariates (age, sex, smoking, drinking, physical activity, blood pressure, lipids, education, marital status, family history, and baseline HbA1c). The “Normal BMI + Low PNI” group serves as the reference. High BMI groups show substantially larger increases in ΔHbA1c compared to normal BMI groups.

To explore the combined effects of BMI and PNI, participants were categorized into four groups based on BMI (<24 vs. ≥24 kg/m^2^, according to the Chinese criteria for overweight/obesity) and PNI (below vs. above the median). Using the “Normal BMI + Low PNI” group as the reference, all other groups showed significantly greater increases in ΔHbA1c after multivariable adjustment (Table S4, Figure 2).

Notably, the two groups with high BMI (≥24 kg/m^2^) exhibited the largest increases in ΔHbA1c, “High BMI + Low PNI” (β = 0.0755, 95% CI: 0.0532–0.0978; *p* < 0.001) and “High BMI + High PNI” (β = 0.0735, 95% CI: 0.0510–0.0960; *p* < 0.001), with nearly identical effect estimates. In contrast, the “Normal BMI + High PNI” group had a smaller but still significant increase (β = 0.0306, 95% CI: 0.0083–0.0529; *p* = 0.007).

These results demonstrate that high BMI is associated with a substantially larger ΔHbA1c increment than PNI alone, and that its association is not appreciably modified by PNI status. PNI contributes a detectable, albeit smaller, independent association primarily in normal-weight individuals.

### 3.5. Joint Continuous Association of BMI and PNI with Glycemic Change

**Figure 3:**
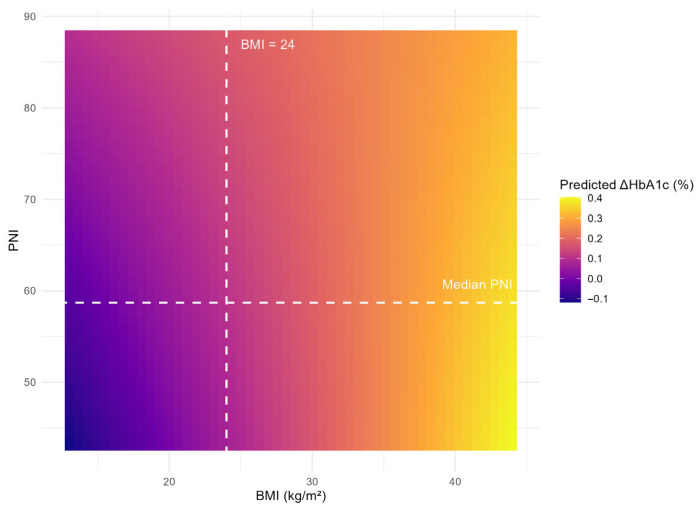
Joint prediction of 3-year glycemic change (ΔHbA1c, %) by BMI and PNI. The heatmap was generated from the fully adjusted linear regression model (Model 3) with all covariates fixed at their means (continuous) or modes (categorical). The color scale represents predicted ΔHbA1c. Vertical and horizontal dashed lines indicate the cutoffs for high BMI (≥24 kg/m^2^) and the median PNI (58.8), respectively. The plot illustrates that BMI is the predominant predictor of glycemic increase, while PNI has a minimal and non-interactive effect.

To visualize the combined continuous effects of BMI and PNI on ΔHbA1c, a prediction heatmap was generated based on the fully adjusted model (Figure 3). The color gradient represents the predicted ΔHbA1c across the range of BMI and PNI values, with all other covariates held constant at their mean or modal values. The dashed lines indicate the cutoffs for high BMI (≥24 kg/m^2^) and the median PNI (58.8).

The heatmap shows that higher BMI is associated with progressively greater ΔHbA1c regardless of PNI level, whereas PNI contributes only a modest increase, most evident at lower BMI values. This continuous visualization further supports the primary finding that BMI is the dominant driver of glycemic deterioration, with PNI exerting a modest independent effect that does not significantly interact with BMI.

## 4. Discussion

In this community-based prospective cohort of 9414 middle-aged and older Chinese adults without diabetes, we examined the associations of baseline BMI and PNI with 3-year glycemic change (ΔHbA1c). Three principal findings emerged. First, BMI showed a **consistent, independent positive** association with ΔHbA1c, stable across models and PNI strata, though with a modest absolute effect size (0.013% per kg/m^2^). Second, PNI also exhibited a small but statistically significant positive association, contrary to our initial hypothesis. Third, joint analysis showed that elevated BMI was the primary contributor to glycemic worsening, while PNI contributed modestly, principally among normal-weight individuals.

### 4.1. BMI and Glycemic Change: Confirming and Extending Prior Evidence

The positive association between BMI and HbA1c progression is well-established [30]. The novelty of the present study does not lie in demonstrating this association per se, but rather in three contributions: (1) simultaneously quantifying the relative contributions of BMI and PNI in a prospective design, providing the first longitudinal evidence of PNI’s independent glycemic association in a non-diabetic community cohort; (2) demonstrating that the BMI–glycemia association operates independently of nutritional–immune status, as assessed by PNI; and (3) identifying that the glycemic signal of PNI is detectable specifically in normal-weight individuals, a subgroup that may benefit from targeted nutritional monitoring [31].

The absolute effect of BMI was modest: each 1 kg/m^2^ increase was associated with only a 0.013% rise in ΔHbA1c. A hypothetical 5 kg/m^2^ increase—an extreme change over three years—would correspond to a 0.065% increment, raising mean HbA1c from 5.62% to approximately 5.69%. The three-year mean ΔHbA1c of 0.35% observed in this cohort was driven by multiple factors, including aging, lifestyle, and baseline glycemic risk, rather than by BMI alone. We therefore refrain from attributing the entirety of observed glycemic change to BMI. The biological pathways through which excess adiposity impairs glycemia—including adipokine-driven insulin resistance, ectopic fat deposition, and low-grade systemic inflammation [32,33,34]—remain operative regardless of PNI status, explaining the stability of the BMI association across quartiles.

### 4.2. PNI and Glycemic Change: An Unexpected Direction and Potential Mechanisms

Contrary to our expectation, higher PNI was associated with modest glycemic deterioration. Several biologically plausible explanations exist. First, in a relatively well-nourished community cohort, a higher PNI may partly reflect overnutrition. Serum albumin is a negative acute-phase reactant [35], and lymphocyte counts can be elevated in low-grade inflammatory states associated with excess adiposity and dietary patterns [36]. Thus, high PNI in this setting may capture a subtle pro-inflammatory milieu rather than optimal nutritional status. Second, the “protein-centric” hypothesis suggests that higher protein intake may augment gluconeogenesis and β-cell insulin demand, potentially accelerating glycemic deterioration in susceptible individuals [37,38]. Prospective studies support a positive link between animal protein intake and diabetes risk [39,40]. Third, the effect size was small (0.002% per unit), and it is possible that residual confounding from unmeasured dietary patterns or inflammatory biomarkers contributes to this association [41,42]. Future studies incorporating detailed dietary assessments and inflammation markers (e.g., hs-CRP, IL-6) are needed to clarify whether the PNI–glycemia relationship is causal or confounded [42,43].

### 4.3. Absence of Interaction

The non-significant BMI × PNI interaction (*p* = 0.431), replicated across PNI quartile strata, suggests that adiposity and nutritional–immune status influence glycemic trajectories through largely independent mechanisms. BMI likely operates through canonical insulin resistance pathways [44], while PNI may reflect nutritional and inflammatory processes that are additive rather than interactive [45,46].

### 4.4. Joint Associations

Cross-classification by BMI and PNI category provided a clinically intuitive stratification. Both high-BMI groups showed ΔHbA1c increments of approximately 0.074–0.076% relative to the normal-BMI/low-PNI reference, irrespective of PNI level—underscoring that in overweight/obese individuals, adiposity dominates the glycemic signal and PNI provides little additional differentiation. Among normal-weight individuals, those with high PNI showed a small additional increment (0.031%), consistent with the independent PNI association described above.

### 4.5. Population Heterogeneity: Normoglycemic vs. Prediabetes

The cohort comprised both normoglycemic (HbA1c < 5.7%, *n* = 3821) and prediabetic (HbA1c 5.7–6.4%, *n* = 5593) participants at baseline [47,48]. Subgroup analyses stratified by glycemic status (Supplementary Materials) showed that the BMI–ΔHbA1c association was present in both subgroups, though with a somewhat larger effect estimate in the prediabetes group, consistent with the notion that individuals closer to the diabetic threshold may be more metabolically vulnerable to the effects of adiposity [49,50]. The PNI association was also detected primarily in the prediabetes subgroup [44,51]. These findings suggest that the associations reported in the main analysis are not entirely driven by one subgroup, but that glycemic status may moderate their magnitude [52].

### 4.6. Comparison with Previous Studies

To our knowledge, this is the first prospective study to simultaneously examine BMI and PNI in relation to longitudinal glycemic change in a non-diabetic population. Prior cross-sectional studies have reported inverse associations between PNI and prevalent diabetes [47,48], which apparently contradicts our longitudinal finding. This discrepancy is interpretively important, as cross-sectional designs are susceptible to reverse causation because diabetes-related catabolism reduces both albumin and lymphocytes, lowering PNI [49]. In our prospective design, all participants were free of diabetes at enrollment, allowing temporal inference. A high baseline PNI preceding glycemic deterioration suggests that the positive PNI–glycemia association reflects antecedent metabolic and nutritional conditions rather than a consequence of disease. Longitudinal studies with longer follow-up are needed to determine whether the early positive PNI association eventually reverses as overt diabetes and catabolism ensue.

### 4.7. Clinical Implications

Weight management remains the most actionable target for glycemic deterioration prevention, even in non-diabetic populations. Although the per-unit effect of BMI on ΔHbA1c is modest, the high prevalence of overweight and obesity (approximately 50% of this cohort had BMI ≥ 24 kg/m^2^) means that even small per-unit associations translate into substantial attributable burden at the population level [31].

PNI is not recommended as a routine screening tool for glycemic risk in general populations given its small effect size. However, in normal-weight individuals—who may otherwise appear metabolically low-risk—a high PNI might warrant closer glycemic monitoring, particularly if accompanied by other risk factors. This observation aligns with the growing recognition that metabolically unhealthy normal-weight individuals represent an underappreciated at-risk group [53,54]. Future intervention studies should examine whether modifying dietary protein intake or addressing low-grade inflammation can influence PNI and downstream glycemic outcomes.

### 4.8. Strengths and Limitations

Strengths include the prospective design, large sample size (*n* = 9414), comprehensive covariate adjustment verified by AIC, exclusion of participants with infections or inflammatory diseases to protect PNI validity, and simultaneous evaluation of BMI and PNI providing novel relative effect estimates.

Limitations include: (1) the observational design precludes causal inference; (2) the 3-year follow-up may be insufficient to capture longer-term glycemic trajectories; (3) dietary quality and inflammatory biomarkers were not available; (4) PNI was measured at a single baseline timepoint and may not reflect longitudinal changes in nutritional–immune status; (5) the findings may not generalize beyond this community-dwelling, middle-aged-to-older Chinese population.

## 5. Conclusions

In this community-based prospective cohort of Chinese adults without diabetes at baseline, BMI showed a consistent and independent positive association with 3-year HbA1c change, although the absolute effect size was modest and the total observed glycemic change reflects contributions from multiple factors beyond BMI. PNI showed a small positive association, contrary to expectation, suggesting that in a relatively healthy community sample, higher PNI may partly reflect subtle pro-glycemic factors rather than representing unambiguous nutritional benefit. The absence of interaction indicates that BMI and PNI operate through largely independent pathways. Joint analyses confirmed that high BMI is the primary contributor to glycemic worsening, while PNI contributes a detectable but small additional signal, principally among normal-weight individuals. The principal novelty of this study lies in prospectively characterizing PNI as a modest, independent glycemic predictor in non-diabetic adults, a finding not previously demonstrated, and in showing that BMI’s glycemic effect is not modified by nutritional–immune status. These results reinforce weight management as the cornerstone of diabetes prevention and call for further investigation into dietary and inflammatory mechanisms underlying the PNI–glycemia relationship.

## Figures and Tables

**Table 1:** Baseline characteristics of the study population.

Characteristic	N = 9414
Age (years)	56.54 (10.33)
Male, *n* (%)	3677 (39%)
BMI (kg/m^2^)	24.29 (3.25)
PNI	58.79 (4.20)
HbA1c (%)	5.62 (0.40)
Systolic BP (mmHg)	133.26 (19.17)
Diastolic BP (mmHg)	80.66 (10.64)
Total cholesterol (mmol/L)	4.91 (0.93)
Triglycerides (mmol/L)	1.59 (1.13)
HDL cholesterol (mmol/L)	1.41 (0.34)
LDL cholesterol (mmol/L)	2.78 (0.84)
Smoking, *n* (%)	2094 (22%)
Alcohol consumption, *n* (%)	1261 (13%)
Physical activity, MET-min/week	1732.53 (1165.68)
Education, *n* (%)	
Low	4460 (47%)
Middle	4282 (45%)
High	672 (7.1%)
Marital status, *n* (%)	8816 (94%)
Family history of T2DM, *n* (%)	929 (9.9%)
Normoglycemic (HbA1c < 5.7%), *n* (%)	3821 (40.6%)
Prediabetes (HbA1c 5.7–6.4%), *n* (%)	5593 (59.4%)
ΔHbA1c (3-year change), %	0.35 (0.62)

Data are presented as mean (SD) for continuous variables and *n* (%) for categorical variables. BMI: body mass index; PNI: prognostic nutritional index; HDL: high-density lipoprotein; LDL: low-density lipoprotein; MET: metabolic equivalent.

**Table 2:** Linear regression models for the association of BMI and PNI with ΔHbA1c.

Variable	Model 1	Model 2	Model 3
BMI (per 1 kg/m^2^)	0.0152 (0.0127, 0.0177) ***	0.0141 (0.0116, 0.0166) ***	0.0134 (0.0108, 0.0160) ***
PNI (per 1 unit)	0.0028 (0.0010, 0.0046) **	0.0026 (0.0008, 0.0044) **	0.0023 (0.0004, 0.0042) *
BMI × PNI	–	–	−0.0002 (−0.0008, 0.0003)
*p* for interaction	–	–	0.431
AIC	–	–	Model 3 AIC lower than Models 1 and 2

Data are presented as β coefficients with 95% confidence intervals (CIs). Model 1: unadjusted. Model 2: adjusted for age and sex. Model 3: adjusted for age, sex, smoking status, alcohol consumption, physical activity, systolic and diastolic blood pressure, total cholesterol, triglycerides, HDL cholesterol, LDL cholesterol, education, marital status, family history of diabetes, and baseline HbA1c. * *p* < 0.05, ** *p* < 0.01, *** *p* < 0.001.

**Table 3:** Association between BMI and ΔHbA1c stratified by PNI quartiles.

PNI Quartile	N	β (95% CI) for BMI	*p*
Q1 (≤55.9)	2369	0.0126 (0.0075, 0.0176)	<0.001
Q2 (56.0–58.7)	2355	0.0151 (0.0104, 0.0198)	<0.001
Q3 (58.8–61.4)	2358	0.0141 (0.0091, 0.0192)	<0.001
Q4 (≥61.5)	2332	0.0126 (0.0070, 0.0182)	<0.001

## Data Availability

The data that support the findings of this study are jointly owned by Fudan University and the Songjiang District Center for Disease Control and Prevention. Due to institutional data governance policies and confidentiality agreements, the raw data are not publicly available. However, data can be accessed upon reasonable request to the corresponding author, subject to approval by the data management committee and execution of a data use agreement.

## Supplementary Materials

The following supporting information can be downloaded at: https://www.mdpi.com/article/10.3390/nu18091459/s1, Figure S1. Restricted cubic spline analysis of BMI and ΔHbA1c. The solid line represents the estimated ΔHbA1c across BMI values, with the shaded area indicating the 95% confidence interval. The histogram at the bottom illustrates the distribution of BMI. p for non-linearity = 0.870. Figure S2. Dose-response relationship: BMI simple slope across PNI levels. The simple slopes of BMI on ΔHbA1c (with 95% confidence bands) are shown at selected percentiles of PNI (10th, 25th, 50th, 75th, and 90th). Slopes were derived from a fully adjusted model that included the BMI × PNI interaction term. All slopes were close to zero, and the confi-dence intervals included the null value, indicating no variation in the effect of BMI across PNI levels. Table S1. Subgroup analysis of the interaction between BMI and PNI on ΔHbA1c. Table S2. Simple slopes of BMI on ΔHbA1c at selected PNI percentiles. Table S3. Mediation analysis of the association between BMI and ΔHbA1c through the TyG index. Table S4. Sensitivity analysis in participants with diabetes at baseline.
