# Supplementary material for: BMI and Prognostic Nutritional Index Are Independently and Positively Associated with Three Year Glycemic Change in Non-Diabetic Adults: A Community-Based Cohort Study
Source: Nutrients. 2026 May 1;18(9):1459. doi: 10.3390/nu18091459 (PMC13165114; doi:10.3390/nu18091459)
Supplement: Supplementary file 1 [file nutrients-18-01459-s001.zip › nutrients-4263149-supplementary.pdf]

Supplementary Analyses

Several supplementary analyses were conducted to assess the robustness of our findings and to explore potential underlying mechanisms.

- **Non-linearity**: Restricted cubic spline analysis revealed no evidence of a non-linear association between BMI and ΔHbA1c (P for non-linearity = 0.870; Figure S1).

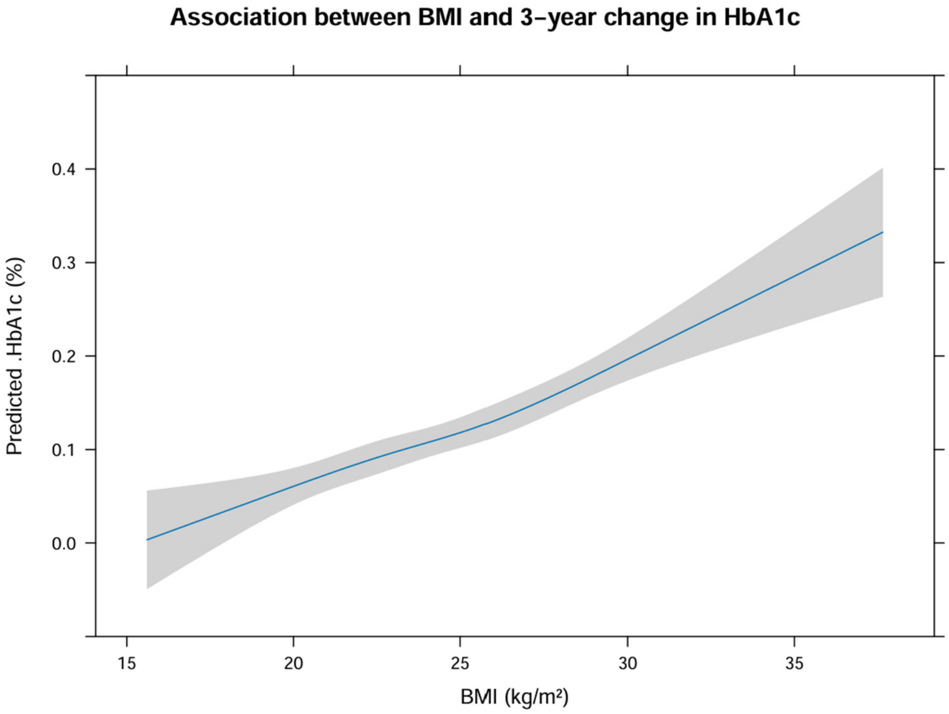

Figure S1. Restricted cubic spline analysis of BMI and ΔHbA1c. The solid line represents the estimated ΔHbA1c across BMI values, with the shaded area indicating the 95% confidence interval. The histogram at the bottom illustrates the distribution of BMI. p for non-linearity = 0.870.

- **Subgroup analyses**: No significant interaction between BMI and PNI was observed across subgroups defined by age, baseline HbA1c, and BMI category (all P for interaction > 0.05; Table S1).

Table S1. Subgroup analysis of the interaction between BMI and PNI on ΔHbA1c.

Subgroup	Level	N	β for interaction (95% CI)	p for interaction
Age group	<60 years	5,281	-0.00007 (-0.00079, 0.00064)	0.841
	≥60 years	4,133	-0.00035 (-0.00120, 0.00050)	0.422
Baseline HbA1c	<5.7%	4,892	-0.00014 (-0.00079, 0.00051)	0.672

	5.7–6.4%	4,522	-0.00020 (-0.00108, 0.00068)	0.654
BMI category	Normal (<24)	4,568	0.00016 (-0.00121, 0.00153)	0.818
	Overweight (24–28)	3,695	0.00154 (-0.00112, 0.00420)	0.256
	Obese (≥28)	1,151	0.00153 (-0.00186, 0.00492)	0.376

All models were adjusted for age, sex, smoking status, alcohol consumption, physical activity, systolic and diastolic blood pressure, total cholesterol, triglycerides, HDL cholesterol, LDL cholesterol, education level, marital status, family history of diabetes, and baseline HbA1c (except when the variable was used as a stratification factor). CI, confidence interval.

- **Dose-response**: Simple slope analysis across PNI percentiles indicated that the effect of BMI on ΔHbA1c did not vary with increasing PNI (Table S2; Figure S2).

Table S2. Simple slopes of BMI on ΔHbA1c at selected PNI percentiles

PNI percentile	PNI value	BMI slope (β)	SE	95% CI	p
10th	53.55	0.00179	0.01486	(-0.02733, 0.03091)	0.904
25th	55.95	0.00127	0.01552	(-0.02914, 0.03169)	0.935
50th	58.70	0.00067	0.01627	(-0.03123, 0.03257)	0.967
75th	61.45	0.00007	0.01703	(-0.03331, 0.03346)	0.996
90th	64.10	-0.00050	0.01776	(-0.03531, 0.03431)	0.977

Simple slopes represent the change in ΔHbA1c (%) per 1 kg/m^2 increase in BMI at specified PNI values, derived from a fully adjusted model that includes the BMI × PNI interaction term. SE, standard error; CI, confidence interval.

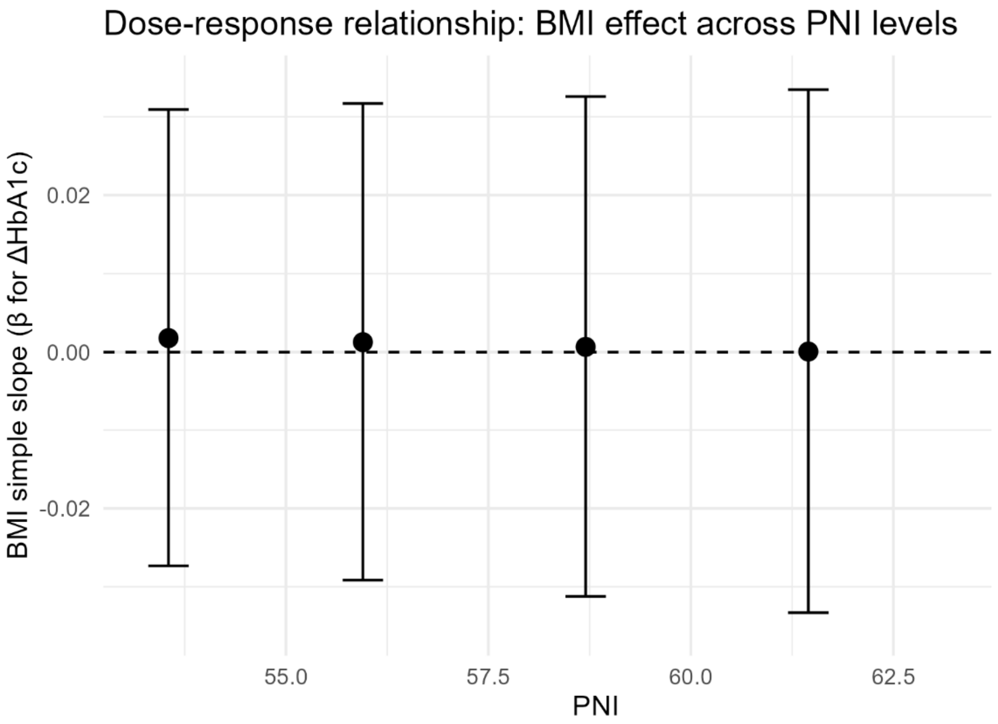

Figure S2. Dose-response relationship: BMI simple slope across PNI levels. The simple slopes of BMI on ΔHbA1c (with 95% confidence bands) are shown at selected percentiles of PNI (10th, 25th, 50th, 75th, and 90th). Slopes were derived from a fully adjusted model that included the BMI × PNI interaction term. All slopes were close to zero, and the confidence intervals included the null value, indicating no variation in the effect of BMI across PNI levels.

- **Mediation analysis**: The association between BMI and ΔHbA1c was not mediated by the TyG index (a surrogate marker of insulin resistance), as the average causal mediation effect was not significant (ACME = 0.00044, 95% CI: −0.00028 to 0.00098; P = 0.186; Table S3).

Table S3. Mediation analysis of the association between BMI and ΔHbA1c through the TyG index.

Effect	Estimate (95% CI)	*p*
ACME (average causal mediation effect)	0.00044 (-0.00028, 0.00098)	0.186
ADE (average direct effect)	0.01311 (0.01013, 0.01635)	<0.001
Total effect	0.01355 (0.01058, 0.01669)	<0.001
Proportion mediated	0.0323 (-0.0207, 0.0780)	0.186

Estimates were based on 1,000 bootstrap simulations. The TyG index was calculated as ln(triglycerides [mg/dL] × fasting glucose [mg/dL] / 2). The model was adjusted for age, sex, smoking status, alcohol consumption, physical activity, systolic and diastolic blood pressure, total cholesterol, HDL cholesterol, LDL cholesterol, education level, marital status, family history of diabetes, and baseline HbA1c.

ACME, average causal mediation effect; ADE, average direct effect; CI, confidence interval.

- **Sensitivity analysis**: In the subgroup of participants with diabetes at baseline (n = 3,245), the interaction between BMI and PNI remained non-significant (P = 0.900; Table S4), and the pattern of joint associations was similar to that observed in the main analysis.

Table S4. Sensitivity analysis in participants with diabetes at baseline.

Population	N	β for BMI × PNI (95% CI)	p
Diabetic at baseline	3,245	0.00023 (-0.00331, 0.00378)	0.897

The model was adjusted for the same covariates as in the main analysis (age, sex, smoking status, alcohol consumption, physical activity, blood pressure, lipid levels, education level, marital status, family history, and baseline HbA1c). CI, confidence interval.
